# *Veronicastrum axillare* Alleviates Ethanol-Induced Injury on Gastric Epithelial Cells via Downregulation of the NF-kB Signaling Pathway

**DOI:** 10.1155/2017/7395032

**Published:** 2017-01-15

**Authors:** Wei-chun Zhao, Yan-shan Xu, Gang Chen, Yan Guo, Dan-yi Wang, Gui-bin Meng

**Affiliations:** ^1^College of Life Sciences, Zhejiang Chinese Medical University, Hangzhou 310053, China; ^2^Department of Chemistry, Simon Fraser University, Burnaby, BC, Canada V5A 1S6

## Abstract

We used human gastric epithelial cells (GES-1) line in an ethanol-induced cell damage model to study the protective effect of* Veronicastrum axillare* and its modulation to NF-*κ*B signal pathway. The goal was to probe the molecular mechanism of* V. axillare* decoction in the prevention of gastric ulcer and therefore provide guidance in the clinical application of* V. axillare* on treating injuries from chronic nephritis, pleural effusion, gastric ulcer, and other ailments. The effects of* V. axillare*-loaded serums on cell viability were detected by MTT assays. Enzyme-linked immunosorbent assay (ELISA) and Real-Time PCR methods were used to analyze the protein and mRNA expression of TNF-*α*, NF-*κ*B, I*κ*B*α*, and IKK*β*. The results showed that* V. axillare*-loaded serum partially reversed the damaging effects of ethanol and NF-*κ*B activator (phorbol-12-myristate-13-acetate: PMA) and increased cell viability. The protein and mRNA expressions of TNF-*α*, NF-*κ*B, I*κ*B*α*, and IKK*β* were significantly upregulated by ethanol and PMA while they were downregulated by* V. axillare*-loaded serum. In summary,* V. axillare*-loaded serum has significantly protective effect on GES-1 against ethanol-induced injury. The protective effect was likely linked to downregulation of TNF-*α* based NF-*κ*B signal pathway.

## 1. Introduction

Gastric ulcer is one of the most common clinical conditions of the digestive system. Globally, more than 14 million people are diagnosed with gastric ulcer annually, and about 4 million people would die from related complications [1]. Human gastric epithelial tissue is in constant turn-over, maintaining a balance between cell death and new cell formation. It has some capacity to repair food-oriented chemical and physical damage [2–4]. However when the damage exceeds the self-repair capacity, the structure and function of the gastric epithelial tissue can be compromised, leading to an imbalance between cell generation and cell death, which in turn can cause gastric inflammation and ulcer.

Alcohol is an indispensable part in many people's life. It is even dubbed as catalyst to build relationships and make deals amongst businessmen. Although alcohol in moderation has health benefits, elevated alcohol consumption has been linked to GI track disorders, and acute GI epithelial tissue alcohol damage is on the rise [5]. Excessive consumption of hard liquor can directly cause gastric epithelial cell damage, resulting in inflammation, congestion, edema, bleeding, erosion, and ulcer in the GI track. Chronic alcoholism has strong ties to GI disorders, atrophic gastritis, and cancer [5, 6]. Therefore, alcohol has been recognized as a key pathogen in gastric epithelial tissue damage. Methods for the prevention and treatment of such damage and deduction of protective mechanisms have been the focus of many research projects [7].

*Veronicastrum axillare* (Sieb. et Zucc) Yamazaki (Scrophulariaceae) has been widely used both alone and in conjugation with other herbs in Chinese folk medicines to treat injuries from chronic nephritis, pleural effusion, gastric ulcer, furunculosis, burns, snake bites, and other ailments [8–13]. Our preliminary studies showed that the water extract of* V. axillare* significantly reduced ethanol-induced gastric damage in a rat model and downregulation of the expression of the key factors of NF-*κ*B signaling pathway [14–19]. In this study we used human GES-1 cell line in an ethanol-induced cell damage model to study the protective effect of* V. axillare* and its modulation to NF-*κ*B signal pathway. The goal was to probe the molecular mechanism of* V. axillare* decoction in the prevention of gastric ulcer and therefore provide guidance in the clinical application of* V. axillare* on treating injuries from chronic nephritis, pleural effusion, gastric ulcer, furunculosis, and other ailments.

## 2. Materials and Methods

### 2.1. Materials and Reagents

*V. axillare* was picked from Lishui in Zhejiang province of China, which was identified as Scrophulariaceae,* V. axillare* plant by Professor Zhensheng Yao (Zhejiang Chinese Medical University). The preparation of high-dose decoction (0.14 g dried plant per mL) followed the protocol of Du [6]. The high-dose decoction was diluted 1 : 1 and 1 : 3 with water to prepare medium-dose and low-dose solutions, respectively. Ranitidine capsule (Shanghai Modern HaSen (Shangqiu) Pharmaceutical Co., Ltd., lot 14071604) was made into 0.18% suspension based on the labeled API content just before use. Other reagents and supplies were obtained from their respective commercial suppliers: absolute ethanol, Hangzhou Shuanglin Chemicals (lot 20140820); GES-1 cell line: immortalized human gastric epithelial cell line, Cancer Hospital Chinese Academy of Medical Sciences; DMEM high glucose culture medium, HyClone; PMA (phorbol-12-myristate-13-acetate), Sigma-Aldrich; NF-*κ*B/TNF-*α* ELISA kit (lot 20150701A) and I*κ*B*α*/IKK*β* ELISA kit (lot 20150801A), Shanghai Yuanye Biotech Ltd.; PrimeScript™ RT Master Mix (RR036A) and SYBR Premix Ex Taq™ II (lot RR820A), Takara.

### 2.2. Equipment

Thermo 3111 CO_2_ incubator (Thermo, USA), TE2000-S inverted phase differential microscope (Nikon, Japan), Milli-Q water purification station (Millipore), 3K15 refrigerated centrifuge (Sigma, Germany), Tanon 2500 gel imaging station (Tianneng Scientific Co. Ltd.), Q5000 micro UV-Vis spectrophotometer (Quawell, USA), StepOnePlus™ Real-Time PCR system (ABI Co.), and Multiskan Flash microplate reader (Thermo, USA) were used.

### 2.3. Drug-Loaded Serum Preparation

20 male SD rats (SPF grade, 200 ± 20 g, Shanghai Sciple Biky Company, certificate SCXK (Hu) 2013-0003) were randomly divided into normal group, Ranitidine group,* V. axillare* high-dose, medium-dose, and low-dose groups, four in each group. The rats were hosted at 23 ± 2°C, 50–70% RH. The rats were daily intragastrically given at 20 mL/kg the following solutions, respectively: 0.9% saline, 0.027 g/kg Ranitidine (equivalent to 3 times the human clinical dose), and* V. axillare* decoction (0.140 g/mL, 0.070 g/mL, and 0.035 g/mL for high-/medium-/low-dose group, resp.). The animals had access to water and food* ad libitum* during the first 13 days and were denied food for the 14th day while they still have free access to water. Two hours after the final dosing, the animals were euthanized by giving 3.0 mL/kg of 10% chloral hydrate subcutaneously, and blood was taken from the abdominal aorta. The blood samples were left at room temperature for 1 h and then centrifuged at 3500 rpm for 10 min. The resulting supernatants were passed through 0.22 *μ*m sterile filters to yield the drug-loaded serums (4-5 mL/rat), which were divided into small aliquots and stored at −20°C.

### 2.4. Cell Subculture

GES-1 cell lines were cultured in 10% FBS supplemented DMEM medium in 25T culturing flasks at 37°C/5% CO_2_, and the medium was changed every other day. When the cells have fully covered the flask bottom, 1 mL of 0.25% Trypsin was added to digest the monolayer and cells were subcultured at 1 : 3 ratio.

### 2.5. *In Vitro* Model

Cultured GES-1 were aliquoted to 10 groups, that is, normal group, model group, Ranitidine group (positive control),* V. axillare* high-/medium-/low-dose groups, PMA group, and* V. axillare* high-/medium-/low-dose + PMA groups. GES-1 culture in its logarithmic growth phase was used to inoculate normal growth medium in a cell cultural plate (six-well plate: 2 mL/well, 1.2 × 10^6^ cells; 96-well plate: 100 *μ*L/well, 4 × 10^4^ cells). After the cells have adhered to the bottom of the wells for 24 h, the mediums were removed, washed twice with PBS, and then replaced with DMEM medium supplemented with 10% drug-loaded rat serum prepared above (drug-loaded DMEM medium). The cells were further cultured for 24 h, and then the mediums in all but the normal group wells were replaced with drug-loaded DMEM medium containing 5% ethanol [3]. After 4 h, cells in wells were used for the subsequent experiments [3].

### 2.6. Cell Viability Assay

MTT stock solution (5 mg/mL, 20 *μ*L/well) was added to GES-1 cell cultures in a 96-well plate prepared in 2.5, and the cells were further cultured for 4 h. The supernatants were removed, and 150 *μ*L of DMSO was added to each well. The optical absorption was measured at 490 nm on a microplate reader.

### 2.7. Measurement of NF-*κ*B, TNF-*α*, I*κ*B*α*, and IKK*β* Protein Expression by ELISA

The supernatants from different groups of GES-1 cell cultures in a 6-well plate described in 2.5 were placed in sterile microcentrifuge tubes and centrifuged at 3000 rpm for 10 min. The supernatants were transferred to new tubes and the amounts of NF-kB, TNF-*α*, I*κ*B*α*, and IKK*β* were measured according to manufacturers' protocols.

### 2.8. Measurement of NF-*κ*B, TNF-*α*, I*κ*B*α*, and IKK*β* mRNA Expression by RT-PCR

After removing the supernatant, the cells remaining in the 6-well plate (described in 2.7) were treated with Trizol reagent to extract the total RNA [20, 21]. A 0.5 *μ*g aliquot of each RNA sample was reverse transcribed with PrimeScript RT Master Mix kit (Takara Co.). A 2 *μ*L aliquot of each reverse transcription product was amplified with SYBR Prime Ex Tag™ II kit following the manufacturer's protocol to obtain target cDNA (primer sequences see Table 1): predenature at 95°C for 30 sec, followed by 40 cycles of 95°C, 5 sec, then 60°C, 30 sec. The relative amounts of NF-*κ*B, TNF-*α*, I*κ*B*α*, and IKK*β* mRNA were calculated with 2^−ΔΔCt^ method, using *β*-actin as the internal standard.

### 2.9. Statistical Analysis

All results were analyzed using SPSS v. 18.0 (IBM) and expressed as average ± standard error ($\overset{-}{x}\pm S$). Intergroup differences were analyzed by one-way ANOVA, and group-wise comparisons were made using Tukey HSD protocol, with *P* < 0.05 as being statistically significant.

## 3. Results

### 3.1. Effect of* V. axillare*-Loaded Serum on Cell Viability after Ethanol Damage

**Figure 1:**
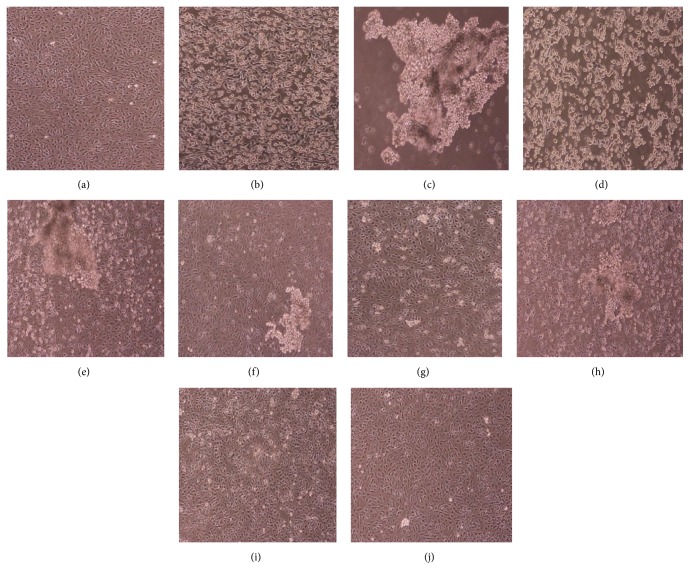
Effect of* Veronicastrum axillare*-loaded serum on cellular morphology of GES-1 injured by ethanol. Note: (a) normal; (b) model; (c) PMA; (d) low dosage of* V. axillare +* PMA; (e) medium dosage of* V. axillare +* PMA; (f) high dosage of* V. axillare +* PMA; (g) Ranitidine; (h) low dosage of* V. axillare*; (i) medium dosage of* V. axillare*; (j) high dosage of* V. axillare*.

**Figure 2:**
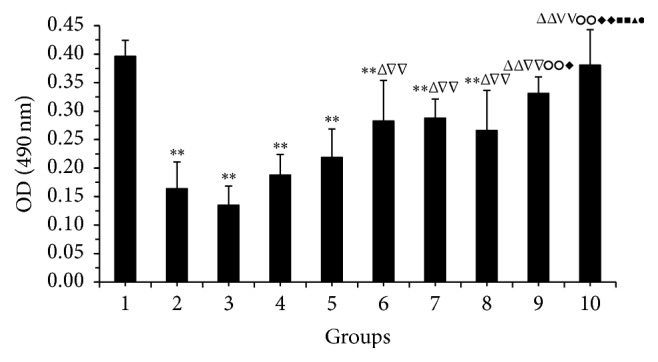
Effect of* Veronicastrum axillare*-loaded serum on cell viability in GES-1 injured by ethanol test by MTT method. Note: 1. normal; 2. model; 3. PMA; 4. low dosage of* V. axillare + *PMA; 5. medium dosage of* V. axillare +* PMA; 6. high dosage of* V. axillare +* PMA; 7. Ranitidine; 8. low dosage of* V. axillare*; 9. medium dosage of* V. axillare*; 10. high dosage of* V. axillare. *Compared with normal, ^*∗*^*P* < 0.05, ^*∗∗*^*P* < 0.01; compared with model, ^△^*P* < 0.05, ^△△^*P* < 0.01; compared with PMA, ^▽▽^*P* < 0.01; compared with low dosage of* V. axillare + *PMA, ^○ ○^*P* < 0.01; compared with medium dosage of* V. axillare + *PMA, ^*◆*^*P* < 0.05, ^*◆◆*^*P* < 0.01; compared with high dosage of* V. axillare + *PMA, ^■■^*P* < 0.01; compared with Ranitidine, ^▲^*P* < 0.05; compared with low dosage of* V. axillare*, ^●^*P* < 0.05.

As seen in Figure 1, normal cells appeared as a dense monolayer that adhered to the bottom and maintained their healthy, cuboidal shapes. In the ethanol model group, most cells had swollen to round shape, and significant numbers dissociated from the wall. Intercellular space increased, connections decreased, and cells separated from each other. In the PMA group, cells formed globular aggregates and almost completely floated to the surface. Cell morphology was even worse than the ethanol model group, suggesting PMA could further stimulate dissociation of cells from each other. In* V. axillare* groups, as the dosing increased, cell morphology improved and eventually got close to normal cells. In low-dose* V. axillare* group, although cells were still swollen, the severity was reduced, and they remained adhered to the culturing dish. In the medium-dose group, cell swelling was greatly reduced, and half of the view field showed normal cell morphology. In the high-dose group, almost no cell swelling was observed. The high-dose group appeared better than the Ranitidine group (positive control). In* V. axillare* + PMA groups, there were also improvements to cell morphology and cell adhesion as the dose increased, but overall cells appeared weaker than the* V. axillare* groups without PMA. Compared with the normal group, cell in the model group, PMA group, and* V. axillare* + PMA groups all showed significant lower viability (Figure 2, *P* < 0.05, *P* < 0.01). Cell viabilities for* V. axillare* high- and medium-dose groups, Ranitidine group, and* V. axillare* high-dose + PMA group were significantly higher than those for the PMA group,* V. axillare* low-dose + PMA group, and* V. axillare* medium-dose + PMA group (*P* < 0.05, *P* < 0.01). Cell viability enhancement in the* V. axillare* high-dose group was much better than those in the* V. axillare* + PMA groups, Ranitidine group, and* V. axillare* low-dose group. This indicated* V. axillare*-loaded serum reduced GES-1 cell damage from PMA treatment.

### 3.2. Effect of* V. axillare*-Loaded Serum on NF-*κ*B, TNF-*α*, I*κ*B*α*, and IKK*β* Level

**Figure 3:**
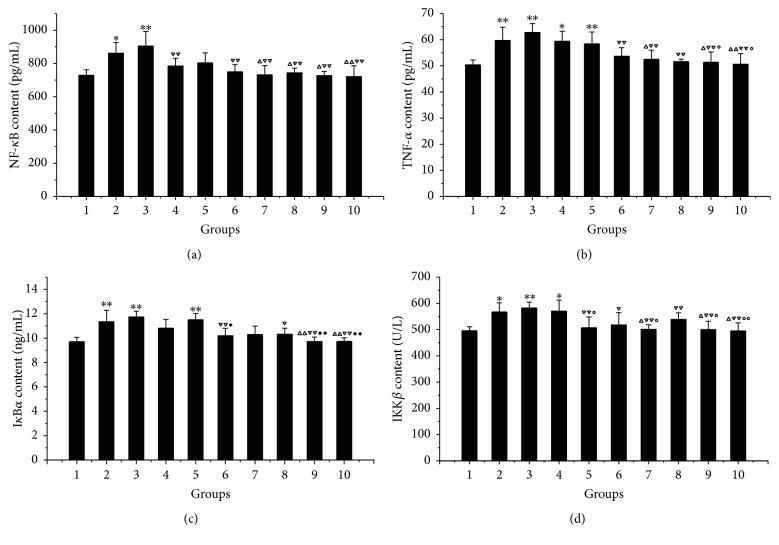
Effect of* Veronicastrum axillare*-loaded serum on NF-*κ*B, TNF-*α*, I*κ*B*α*, and IKK*β* content in GES-1 injured by ethanol. Note: 1. normal; 2. model; 3. PMA; 4. low dosage of* V. axillare + *PMA; 5. medium dosage of* V. axillare +* PMA; 6. high dosage of* V. axillare + *PMA; 7. Ranitidine; 8. low dosage of* V. axillare*; 9. medium dosage of* V. axillare*; 10. high dosage of* V. axillare. *Compared with normal, ^*∗*^*P* < 0.05, ^*∗∗*^*P* < 0.01; compared with model, ^△^*P* < 0.05, ^△△^*P* < 0.01; compared with PMA, ^▽^*P* < 0.05, ^▽▽^*P* < 0.01; compared with low dosage of* V. axillare + *PMA, ^○^*P* < 0.05, ^○ ○^*P* < 0.01; compared with medium dosage of* V. axillare + *PMA, ^*◆*^*P* < 0.05, ^*◆◆*^*P* < 0.01.

As shown in Figure 3, when compared with the normal group, the NF-*κ*B, TNF-*α*, I*κ*B*α*, and IKK*β* levels in the model group and the PMA group all increased (*P* < 0.05, *P* < 0.01). Compared with the model group,* V. axillare* groups and Ranitidine group had significant downregulation of NF-*κ*B, TNF-*α*, I*κ*B*α*, and IKK*β* (*P* < 0.05, *P* < 0.01); the effects in the* V. axillare* groups tend to be dose-dependent, with the high-dose group being the most significant.* V. axillare* high-dose + PMA group,* V. axillare* groups, and Ranitidine group all had significant inhibition on the expression of NF-*κ*B, TNF-*α*, I*κ*B*α*, and IKK*β* when compared with the PMA group (*P* < 0.05, *P* < 0.01). The inhibition of TNF-*α* was stronger in the* V. axillare* high- and medium-dose groups compared with that of* V. axillare* low-dose + PMA group (*P* < 0.05, Figure 3(b)). The downregulation of IkB*α* in the* V. axillare* high- and medium-dose groups and* V. axillare *high-dose + PMA group was more significant than that of* V. axillare *medium-dose + PMA group (*P* < 0.05, *P* < 0.01; Figure 3(c)). The downregulation of IKK*β* in the* V. axillare* high- and medium-dose groups,* V. axillare* medium-dose + PMA group, and Ranitidine group was stronger than that of the* V. axillare* low-dose + PMA group (*P* < 0.01, *P* < 0.05; Figure 3(d)).

### 3.3. Extraction of Total RNA and Analysis of the DNA Amplification Product

**Figure 4:**
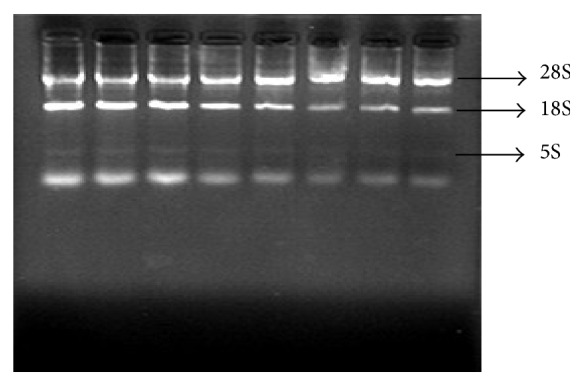
RNA integrity check: nondenaturing agarose gel electrophoresis.

**Figure 5:**
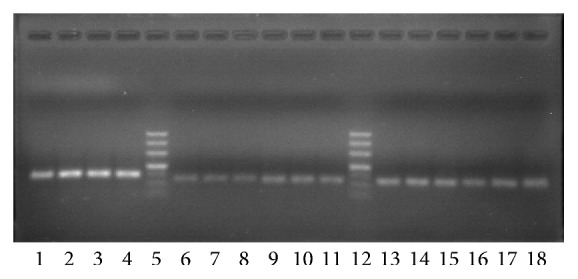
Agarose gel electrophoresis of qRT-PCR products. Note: Lanes 5 and 12: DL500 DNA Ladder Marker (500, 400, 300, 200, 150, 100, and 50 bp in turn from the top down). Lanes 1–4: *β*-actin (144 bp). Lanes 6–8: NF-*κ*B (127 bp). Lanes 9–11: TNF-*α* (119 bp). Lanes 13–15: I*κ*B*α* (116 bp). Lanes 16–18: IKK*β* (118 bp).

The extracted RNA was subjected to nondenaturing agarose gel electrophoresis (2%), and the 5S, 18S, and 28S RNA bands were well defined, proving the integrity of the extracted RNA (Figure 4). Electrophoresis of the PCR products and subsequent fluorescent quantification showed the PCR products were uniform and in agreement with the expected product size, indicating NF-*κ*B, TNF-*α*, IkB*α*, and IKK*β* gene and *β*-actin primers participated in the reactions and the PCR amplifications were successful (Figure 5).

### 3.4. Effect of* V. axillare*-Loaded Serum on the Expression of NF-*κ*B, TNF-*α*, I*κ*B*α*, and IKK*β* mRNA

**Figure 6:**
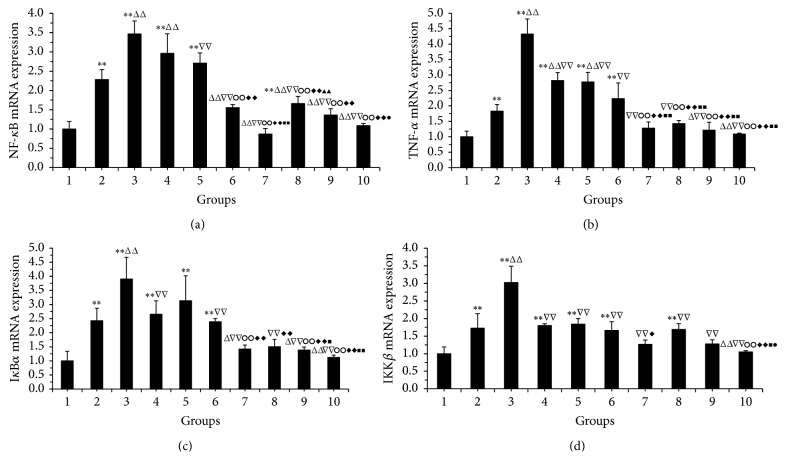
Effect of* Veronicastrum axillare*-loaded serum on NF-*κ*B, TNF-*α*, I*κ*B*α*, and IKK*β* mRNA in GES-1 injured by ethanol. Note: 1. normal; 2. model; 3. PMA; 4. low dosage of* V. axillare + *PMA; 5. medium dosage of* V. axillare +* PMA; 6. high dosage of* V. axillare + *PMA; 7. Ranitidine; 8. low dosage of* V. axillare*; 9. medium dosage of* V. axillare*; 10. high dosage of* V. axillare. *Compared with normal, ^*∗∗*^*P* < 0.01; compared with model, ^△^*P* < 0.05, ^△△^*P* < 0.01; compared with PMA, ^▽▽^*P* < 0.01; compared with low dosage of* V. axillare + *PMA, ^○ ○^*P* < 0.01; compared with medium dosage of* V. axillare + *PMA, ^*◆*^*P* < 0.05, ^*◆◆*^*P* < 0.01; compared with high dosage of* V. axillare + *PMA, ^■^*P* < 0.05, ^■■^*P* < 0.01; compared with Ranitidine, ^▲▲^*P* < 0.01; compared with low dosage of* V. axillare*, ^●^*P* < 0.05.

Figure 6 showed that the model group and PMA group had upregulation of NF-*κ*B, TNF-*α*, I*κ*B*α*, and IKK*β* mRNA compared with the normal group (*P* < 0.01). Moreover, the upregulation of NF-*κ*B in the PMA group was significantly higher than that of the model group (*P* < 0.01, Figure 6(a)), indicating the activation of NF-*κ*B by PMA. The expression of NF-*κ*B, TNF-*α*, I*κ*B*α*, and IKK*β* mRNA were significantly downregulated by high/medium/low dose of* V. axillare* and Ranitidine (*P* < 0.05, *P* < 0.01; Figures 6(a) and 6(c)). Compared with the PMA group, the expression of NF-*κ*B, TNF-*α*, I*κ*B*α*, and IKK*β* mRNA was significantly reduced in* V. axillare* groups,* V. axillare* + PMA groups, and Ranitidine group (*P* < 0.01; Figures 6(a)–6(c)). Compared with the* V. axillare* high-dose + PMA group,* V. axillare *high-dose group further reduced the expression of TNF-*α*, I*κ*B*α*, and IKK*β* mRNA (*P* < 0.05, *P* < 0.01; Figures 6(b)–6(d)), and the downregulation of NF-*κ*B and IKK*β* was more than that of the* V. axillare* low-dose group (*P* < 0.05; Figures 6(a) and 6(d)).

## 4. Discussion

Alcoholic gastric epithelial injury refers to conditions caused by excessive alcohol consumption that induced damage to the gastric epithelial tissues (such as gastric ulcer and gastrorrhagia). The clinical symptoms include abdominal pain, fullness, vomiting, and heartburn [3]. The injury caused by alcohol is typically exerted by inducing epithelial cell damage and apoptosis. Our preliminary* in vivo* results showed that the water extract of* V. axillare *significantly inhibited ethanol-induced gastric epithelial cell damage [14, 15, 19]. The ulcer inhibition rates of low, middle, and high dosage (0.7 g/kg, 1.4 g/kg, and 2.8 g/kg) group were 47.9%, 71.4%, and 89.4%, respectively [15]. The current study used MTT assay to examine the effect on cell viability under ethanol assault by pretreatment of GES-1 with* V. axillare*-loaded serum. The results showed that* V. axillare*-loaded serum had protective effects and reduced the damage caused by ethanol. This is the first evidence at the cellular level that* V. axillare*-loaded serum is effective against damage induced by ethanol as a model of alcoholic gastric ulcer [14–19].

Alcohol can cause gastric epithelial injury by inducing apoptosis through the TNF-*α* pathway and through the formation of ROS, which causes cellular damage through oxidative stress [3]. At the same time, the expression of TNF-*α* is under the control of NF-*κ*B signal pathway. NF-*κ*B signaling pathway is involved in controlling the gene expression of multiple factors and plays an important role in immune response, inflammation, stress response, cell apoptosis, cancer, and ontogenetic development [22–24]. Excessive activation of the NF-*κ*B pathway will trigger body's specific and nonspecific immune response and cause tissue damage and organ dysfunction. The three key factors in the NF-*κ*B pathway are I*κ*B*α*, IKK*β*, and NF-*κ*B [25]. When IKK*β* is activated by inter- or intracellular stimulations, it causes I*κ*B*α* phosphorylation and ubiquitination. Ubiquitinated I*κ*B*α* can be degraded by a 28S small proteasome, which causes the nucleic acid binding domain in NF-*κ*B to be exposed, and the translocation of NF-*κ*B into the nucleus. This in turn will change the chemical structure of I*κ*B, causing a series of biological responses, thus controlling the gene expression of TNF-*α* [17, 26]. Studies have shown that Tong Xin Luo Capsule can protect the retina in a mouse diabetic model by downregulating I*κ*B*α*, IKK*β*, and NF-*κ*B expressions [27]. Severe liver damage was observed in IKK*β*-knockout mice, and IL-1*β* and TNF-*α* induced NF-*κ*B activity was significantly reduced [28]. An earlier* in vivo *study has shown that the protective effect of* V. axillare* on ethanol-induced gastric ulcer of SD rats is intimately linked to TNF-*α* mediated NF-*κ*B signaling pathway [15, 17]. Compared with the ethanol injury model group, the expressions of TNF-*α*, NF-*κ*B, TNFR-1, RIP1, I*κ*B*α*, and IL-1*β* in rats gastric tissue are downregulated by* V. axillare*. The current study has shown that, in the model group, the mRNA expression of NF-*κ*B, TNF-*α*, I*κ*B*α*, and IKK*β* of GES-1 cell lines increased significantly, and the protein concentrations of them in GES-1 supernatant also increased remarkably, while different doses of* V. axillare* downregulated these factors in GES-1 and supernatant to various degrees. Those indicated that* V. axillare* likely exerted the protective effect on GES-1 against ethanol damage through the NF-*κ*B pathway by downregulating the expressions of NF-*κ*B, TNF-*α*, I*κ*B*α*, and IKK*β*. The concentrations of NF-*κ*B, I*κ*B*α*, and IKK*β* in supernatant increased after the GES-1 were damaged by 5% ethanol. This increase was likely due to increased cell membrane permeability from the swelling and cell damage, although there is possibility that these factors are actively secreted. Further studies will be needed to discern the exact mechanism.

PMA can activate protein kinase C (PKC), thus activating NF-*κ*B [29]. Under resting conditions, NF-*κ*B and I*κ*B exist as an inactive complex in the cytoplasm. When cells are stimulated by a PKC agonist, PKC is activated, with the concomitant activation of IKK*β*. IKK*β* can act as the kinase of I*κ*B to cause its phosphorylation and dissociation from NF-*κ*B. I*κ*B*α* contains a gene promoter domain that binds NF-*κ*B, the binding of which causes I*κ*B*α* gene expression to increase rapidly. At the same time, the dissociated NF-*κ*B is activated to display the transcription modulating activity [30–34]. The gene of the proinflammatory cellular factor TNF-*α* has a *κ*B-binding domain. When NF-*κ*B is activated, it will bind to the *κ*B domain and promote its transcription, thus aggravating the inflammation [35, 36]. Study has shown that, with increasing concentration of PMA, the concentration of NF-*κ*B protein in astrocytes also increased [37]. In another study, the damage to myocytes from Doxorubicin was linked to the overactivation of NF-*κ*B and TNF-*α* [38]. This study showed that the expressions of NF-*κ*B, TNF-*α*, I*κ*B*α*, and IKK*β* in the PMA group were significantly higher than those of the normal group and the model group. This may indicate that when PMA activated PKC, it also activated IKK*β*. This would cause the phosphorylation of I*κ*B*α* and its dissociation from NF-*κ*B. The dissociated NF-*κ*B displayed TNF-*α* modulating activity, which affected the NF-*κ*B signaling pathway. Compared with the PMA group,* V. axillare* high-dose + PMA group had significantly lower NF-*κ*B, TNF-*α*, I*κ*B*α*, and IKK*β* levels, which indicated* V. axillare* inhibited the activation of NF-*κ*B pathway by PMA, and in turn supported that* V. axillare* protected GES-1 from alcohol damage by blocking the NF-*κ*B pathway.

Chinese herbal medicines and their active compounds have become an active research field because of their advantages of multitarget, multichannel activities and less recurrence and play an irreplaceable role in the treatment of gastric ulcer. Our preliminary studies showed that the decoction of* V. axillare* can significantly reduce the acute gastric mucosal injury in SD rats induced by ethanol, improving the pathological state of the gastric mucosa, and regulating the secretion of gastric juice and gastric acid [14]. Compared with the ethanol injury model group, the concentrations or activities of multiple protective factors such as NO [12], iNOS [12], superoxide dismutase (SOD) [10], cyclooxygenase 2 (COX2) (unpublished data), prostaglandin E2 (PGE2) (unpublished data), and aquaporins (AQP1) [14] in rat gastric epithelial tissues are upregulated by pretreatment of* V. axillare*, while the activities of pepsin [14] and the expression of endothelin 1 (ET-1) [11], AQP3 [14], and AQP4 [14] are downregulated. The concentrations of EGF [10] and VEGF [12] between the ethanol-injured model group and* V. axillare* group are not significantly different. In this experiment, Ranitidine was used as the positive control. This is because Ranitidine is a commonly used drug in the treatment of gastric ulcer. Ranitidine is a histamine H2 receptor antagonist (H2RA). It can effectively inhibit the secretion of gastric acid caused by histamine, pentapeptide gastrin, and the stimulation of food and reduce the activity of pepsin. Our prior results showed that Ranitidine can significantly reduce ethanol-induced gastric ulcer, increase the level of PGE2, and upregulate the expression of COX-2 protein and mRNA. But the antiulcer effect of Ranitidine was weaker than* V. axillare*. This may be related to their different antiulcer mechanisms. As the antiulcer mechanism of* V. axillare* is not clear, other antiulcer drugs, such as Omeprazole (a proton pump inhibitors, PPIs), Colloidal Bismuth Subcitrate (gastric mucosal protective agent), Dexamethasone (anti-inflammatory drugs), Weikangling Capsule (Chinese medicine), could be included as positive controls in further studies.

In summary,* V. axillare*-loaded serum significantly protected GES-1 against ethanol-induced damage. This effect is likely linked to the downregulation of the expression of NF-*κ*B, TNF-*α*, I*κ*B*α*, and IKK*β*. The exact mechanism remains to be elucidated.

## Figures and Tables

**Table 1:** Forward and reverse primers for *β*-actin, NF-*κ*B, TNF-*α*, I*κ*B*α*, and IKK*β*.

	Forward primer 5′–3′	Reverse primer 5′–3′	Fragment (bp)
*β*-actin	CCTGGCACCCAGCACAAT	GGGCCGGACTCGTCATAC	144
NF-*κ*B	ACTGTGAGGATGGGATCTGC	TCTGTCATTCGTGCTTCCAG	127
TNF-*α*	GGCGTGGAGCTGAGAGATAA	GTGTGGGTGAGGAGCACAT	119
I*κ*B*α*	CCACTCCATCCTGAAGGCTA	CATTGACATCAGCACCCAAG	116
IKK*β*	CCTGGTAGAACGGATGATGG	GTACAGCTCCCTTGCTTGCT	118
